# Transcriptomic Analysis of a Tertiary Relict Plant, Extreme Xerophyte *Reaumuria soongorica* to Identify Genes Related to Drought Adaptation

**DOI:** 10.1371/journal.pone.0063993

**Published:** 2013-05-23

**Authors:** Yong Shi, Xia Yan, Pengshan Zhao, Hengxia Yin, Xin Zhao, Honglang Xiao, Xinrong Li, Guoxiong Chen, Xiao-Fei Ma

**Affiliations:** 1 Key Laboratory of Stress Physiology and Ecology in Cold and Arid Regions, Department of Ecology and Agriculture Research, Cold and Arid Regions Environmental and Engineering Research Institute, Chinese Academy of Sciences, Lanzhou, People’s Republic of China; 2 Key Laboratory of Eco-hydrology and of Inland River Basin, Cold and Arid Regions Environmental and Engineering Research Institute, Chinese Academy of Sciences, Lanzhou, People’s Republic of China; 3 Shapotou Desert Research and Experiment Station, Cold and Arid Regions Environmental and Engineering Research Institute, Chinese Academy of Sciences, Lanzhou, People’s Republic of China; Auburn University, United States of America

## Abstract

**Background:**

*Reaumuria soongorica* is an extreme xerophyte shrub widely distributed in the desert regions including sand dune, Gobi and marginal loess of central Asia which plays a crucial role to sustain and restore fragile desert ecosystems. However, due to the lacking of the genomic sequences, studies on *R. soongorica* had mainly limited in physiological responses to drought stress. Here, a deep transcriptomic sequencing of *R. soongorica* will facilitate molecular functional studies and pave the path to understand drought adaptation for a desert plant.

**Methodology/Principal Findings:**

A total of 53,193,660 clean paired-end reads was generated from the Illumina HiSeq™ 2000 platform. By assembly with Trinity, we got 173,700 contigs and 77,647 unigenes with mean length of 677 bp and N50 of 1109 bp. Over 55% (43,054) unigenes were successfully annotated based on sequence similarity against public databases as well as Rfam and Pfam database. Local BLAST and Kyoto Encyclopedia of Genes and Genomes (KEGG) maps were used to further exhausting seek for candidate genes related to drought adaptation and a set of 123 putative candidate genes were identified. Moreover, all the C_4_ photosynthesis genes existed and were active in *R. soongorica*, which has been regarded as a typical C_3_ plant.

**Conclusion/Significance:**

The assembled unigenes in present work provide abundant genomic information for the functional assignments in an extreme xerophyte *R. soongorica*, and will help us exploit the genetic basis of how desert plants adapt to drought environment in the near future.

## Introduction

Understanding the genetic basis of how organisms adapt to climate change is one of the most challenging tasks [1], [2]. Although many attempts have exploited the adaptation mechanism in the species with known genome sequences, the molecular basis of adaptation in the non-genomic species is still poorly understood [1], [2], especially in the plants from arid regions which contain plenty of potential genetic resources for ecology engineering. As pioneer and constructive species in all kinds of desert ecosystems, *Reaumuria* plants play important roles to sustain fragile desert ecosystems by keeping the vital process of the transport of energy and substances [3]–[5], and preventing from wind erosion, sand drifting and the further desertification of these regions [3], [6]–. These plant species were widely used as fine pioneer plants in the restoration of degraded ecosystems with natural rainfall [9] and in the sustainable development of arid regions due to their extreme tolerance to saline-alkaline conditions [10]–[12].

**Figure 1:**
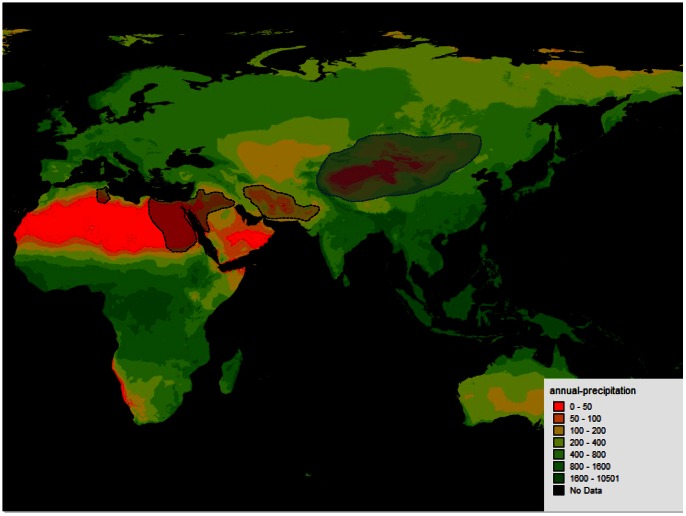
Distribution of *R. soongorica* in northern China. The blue shadow indicates the *Reaumuria* distribution in China and neighboring districts. The black shadow indicates the *Reaumuria* distribution in North Africa and Central Asia.

*Reaumuria* (Tamaricaceae) plants are perennial xeric shrubs, and all the 12 species classified in this genus were distributed in the arid regions from North Africa, Asia, and South Europe, among which four species including *R. soongorica*, *R. alternifolia*, *R. kaschgarica*, and *R. trigyna* were found in China (www.eflora.org, Figure 1). *R.* *soongorica* (2n = 22 with 778 Mb genome size [13]) is one of the constructive and dominant species in kinds of desert ecosystems in central Asia, such as Taklamakan, Gurbantunggut, Kumtag, Badain Jaran, Qaidam, South Russia, South Mongolia and Tenger deserts, and Mu Us, Ulan Buh and Horqin sandy lands, and marginal Loess [14], [15] (Figure 1). Desertification of these regions is getting worse due to accelerating global climate change and human activity [14], [16]. *R. soongorica* has undergone desertification of Asia which initiated at least 22 million years ago according to the palaeomagnetic measurements and fossil evidence [17]. During the process of adaptation to desertification, *R. soongorica* has evolved specific traits including extremely thick cuticle, hollow stomata, specialized leaf shape, deep root system, and effective physiological mechanisms such as reduced transpiration rate, increased water use efficiency, and maintaining the stem vigor to survive desiccation by leaf abscission [7], [18]–[21]. Much effort has been made in *R. soongorica* to elucidate the mechanism of drought adaptation during last decade, however, due to paucity of genomic information, most of the previous studies have limited to its physiological characteristics [21]–[27]. Little work had focused on the genetic diversity based on neutral markers (RAPD [28], ISSR [29], [30] and cpDNA [31]). However, all these studies failed to dissolve the adaptive evolution of *R. soongorica*. Therefore, transcriptome sequences are in urgent to supply sufficient functional genomic information to address systemically the genetic mechanisms of drought adaptation of *R. soongorica*.

In this study, the *R. soongorica* transcriptome was sequenced by the Illumina paired-end sequencing technology (Illumina HiSeq™ 2000 platform). A total of 4.8 gigabases raw data was assembled into 173,700 contigs and further constructed into 77,647 unigenes (mean length = 677 bp, N50 = 1109 bp). Moreover, 123 unigenes were identified to be potentially involved in drought adaptation. To our surprise, all the C_4_ photosynthesis genes were existed and active in *R. soongorica* which has been regarded as a typical C_3_ plant [32]. The *R. soongorica* transcriptomic information provides a prime reference point for the subsequent exploitation of this important genetic resource and will facilitate to unravel the mechanism of adaptation to extreme arid environment.

## Results

### Sequencing and *de novo* Assembly

To obtain a global overview of the *R. soongorica* transcriptome, a pooled cDNA library representing the inflorescences, leaves, and seedlings was constructed, and then sequenced on the Illumina HiSeq™ 2000 platform. A total of 4.8 gigabases dataset was generated from 53,193,660 clean paired-end reads with length of 90 bps and Q20 over 96% (Table 1). This suggested that the sequencing output and quality were good enough for further analysis.

**Table 1:** Summary of *de novo* sequence assembly for *Reaumuria soongorica*.

Item	Number
Total Raw Reads	57,745,560
Total Clean Reads	53,193,660
Total Clean Nucleotides (nt)	4,787,429,400
Q20 percentage (%)	96.32
N percentage (%)	0.01
GC percentage (%)	45.52
Number of contigs	173,700
Shortest contig (bp)	100
Longest contig (bp)	7561
N50 of contigs (bp)	532
Mean length of contig (bp)	321
Number of unigenes	77,647
Shortest unigene (bp)	200
Longest unigene (bp)	7546
N50 of unigenes (bp)	1109
Mean length of unigene (bp)	677

A total of 173,700 contigs with average length of 321 bp and an N50 length of 532 bp were assembled by Trinity program [33]. There were 103,541 contigs (59.61%) ranged from 100 to 200 bp, 67,087 contigs (38.62%) ranged from 201 bp to 2000 bp, and 3,072 contigs (1.77%) longer than 2 kb (Figure S1A). After further clustering and assembly, a total of 77,647 unigenes was generated with average length of 677 bp (200 to 7,546 bp) and N50 length of 1109 bp (Figure S1B). About 40% (30,680) unigenes were longer than 500 bp, among which 5.66% (4,392) unigenes were more than 2000 bp.

### Sequence Annotation

**Figure 2:**
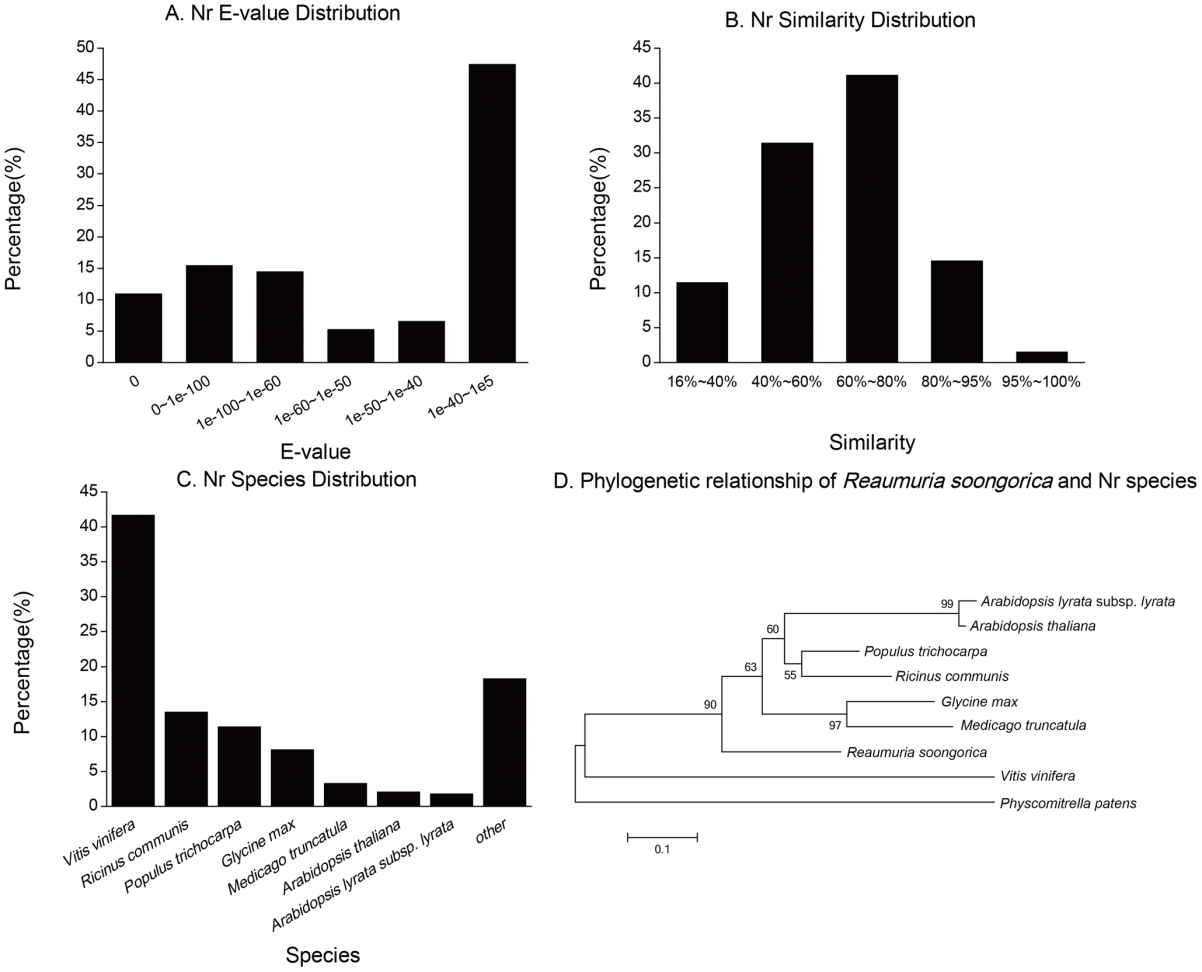
Characteristics of similarity search of unigenes against Nr databases. (A) The E-value distribution of Nr annotation results. (B) The similarity distribution of Nr annotation results. (C) The species distribution of Nr annotation results. (D) The phylogenetic relationship of *Reaumuria soongorica* and Nr species. Phylogenetic tree constructed by the Neighbor-Joining method for the internal transcribed spacer (ITS) sequences. *Physcomitrella patens* was included as an out-group. Neighbor-Joining was consensus tree used 1000 bootstrap replicates. The number represents the percentage of bootstrap values.

Several complementary approaches were utilized to validate and annotate the assembled unigenes. The unigenes were first aligned to the National Center for Biotechnology Information (NCBI) non-redundant protein (Nr) database, non-redundant nucleotide sequence (Nt) database, and the Swiss-Prot protein database with E-values less than 1e-5. Among the 77,647 unigenes, 41,582 (53.55%), 28,197 (36.31%) and 25,297 (32.58%) unigenes were significantly matched to the known genes in the Nr, Nt and Swiss-Prot protein databases, respectively (Table S1 and Table 2). The E-value distribution of the top hits in the Nr database showed that 46.01% of the sequences were mapped to the known genes in plants with best hits (E-value<1e-50, mean identity is 69.54%), and approximately 16% of unigenes can hit deposited sequences with similarity over 80% (Figure 2A and 2B). About 82% of annotated unigenes can be assigned with a best score to the sequences from the top seven species, i.e., *Vitis vinifera* (41.65%), *Ricinus communis* (13.46%), *Populus trichocarpa* (11.39%), *Glycine max* (8.12%), *Medicago truncatula* (3.29%), *Arabidopsis thaliana* (2.08%) and *A. lyrata* subsp. *lyrata* (1.78%) (Figure 2C). Interestingly, the phylogenetic relationship based on the internal transcribed spacer (ITS) also showed *R. soongorica* in among the other rosids species firstly diverged from *V. vinifera*, though the bootstrap value of their relationship is below 50% (Figure 2D).

**Table 2:** Summary of sequence annotation for *Reaumuria soongorica*.

Database	Nr	Nt	Swiss-Prot	GO	COG	KEGG	Rfam	Pfam
Hit numbers (percentage)	41582 (53.55%)	28197 (36.31%)	25297 (32.58%)	29666 (38.20%)	14987 (19.30%)	23569 (30.35%)	42 (0.05%)	173 (0.22%)
Annotated numbers (percentage)	42839 (55.17%)						215 (0.28%)	
Total annotated numbers(percentage)	43054 (55.45%)							

#### GO and COG classification

**Figure 3:**
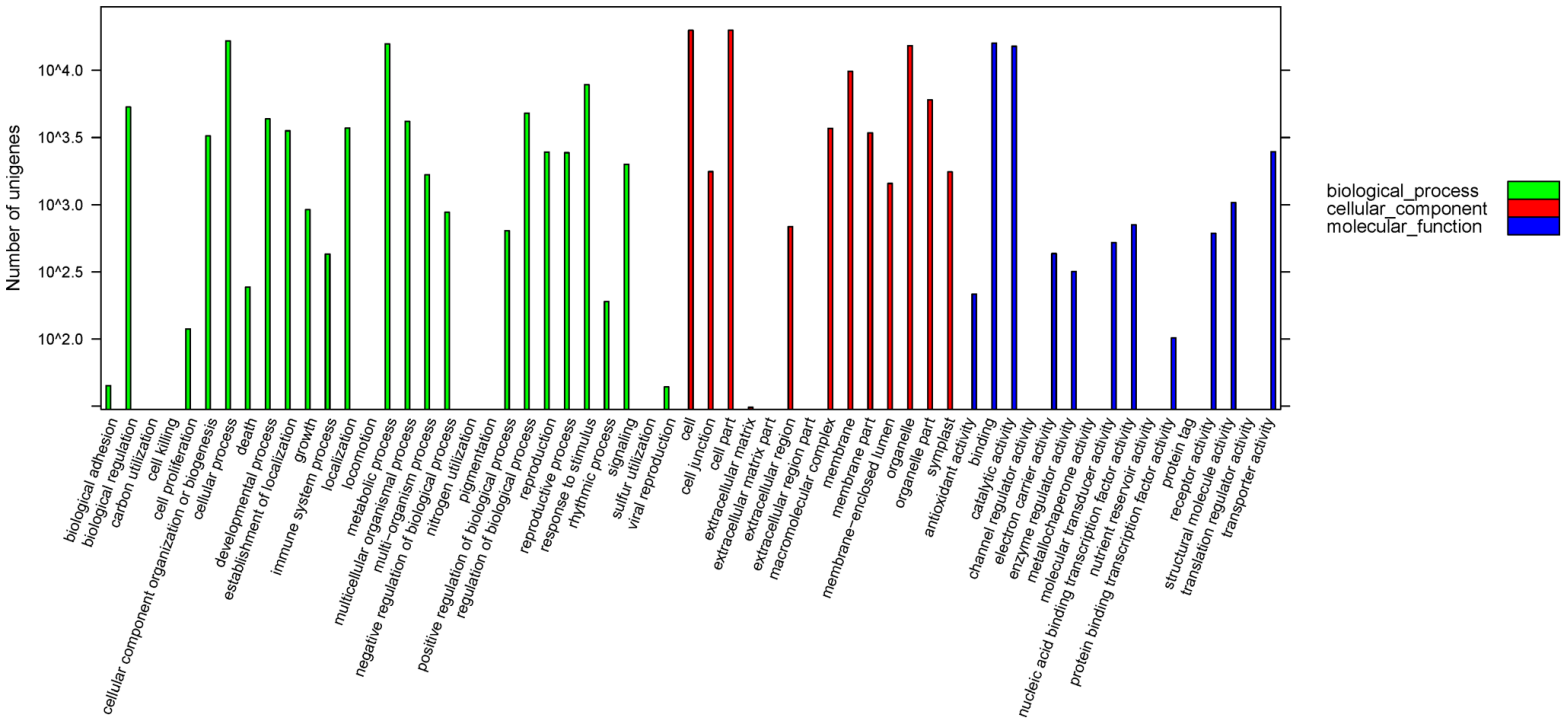
Gene Ontology classification of assembled unigenes. Total 29,666 unigenes were categorized into three main categories: Biological process (81,460, 40.21%), Cellular component (83,650, 41.29%) and Molecular function (37,497, 18.51%).

To identify functional categories among the 77,647 unigenes, all the best BLAST hits were input into the Gene Ontology (GO) Software Blast2GO for GO functional enrichment analysis by performing Fisher’s exact test [34], [35]. In total, 38.21% of unigenes (29,666) could be assigned to gene ontology classes with 202,607 functional terms (Figure 3, Table S2). Interestingly, cellular process (*p* = 0.007), metabolic process (*p* = 0.007) and response to stimulus (*p* = 0.009) are strong significantly overrepresented in the 29 biological process GO groups.

**Figure 4:**
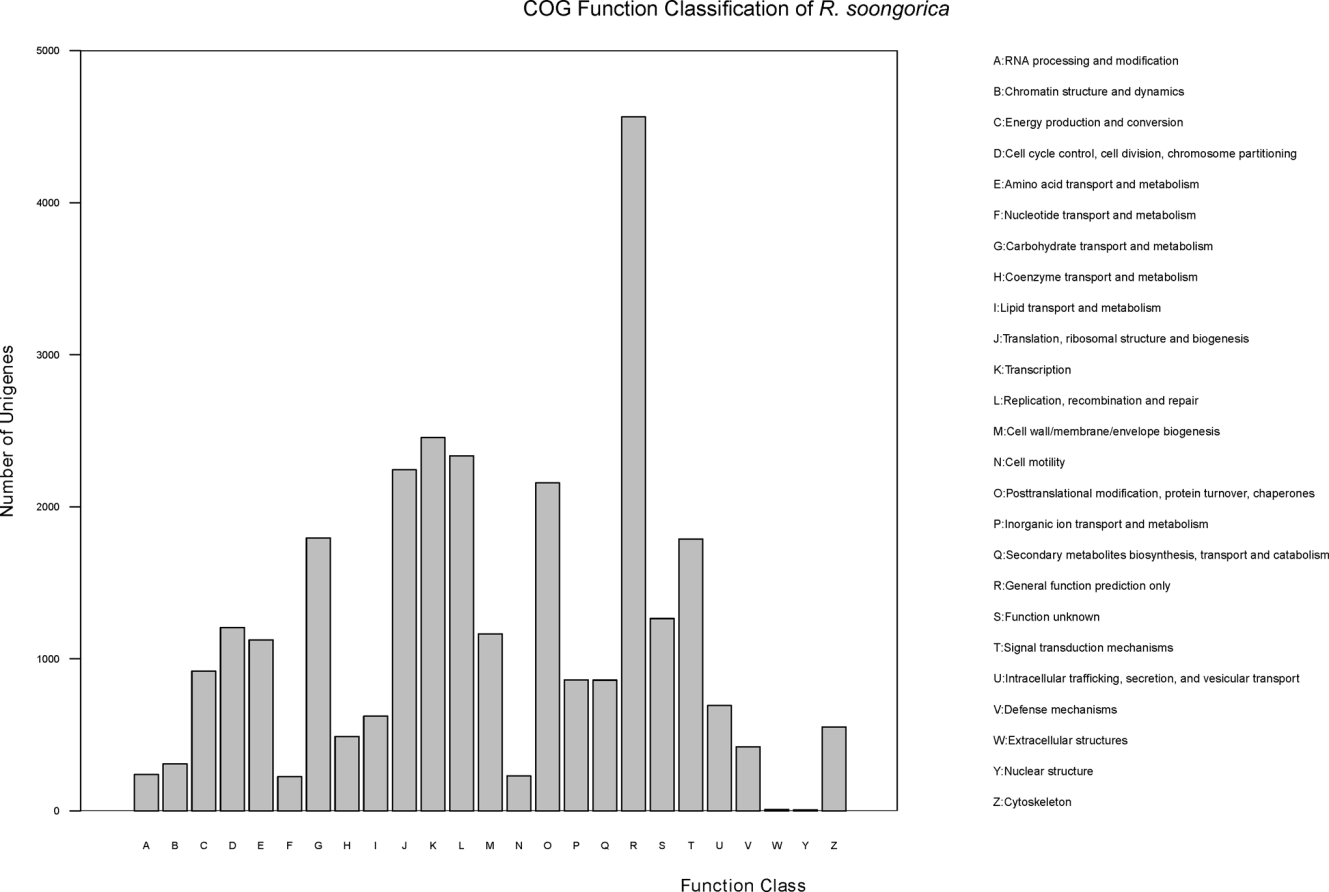
COG function classification. Total 14,987 unigenes were assigned to 25 COG classifications. The largest cluster was for general function prediction only (4,564, 15.99%), followed by transcription (2,455, 8.60%), replication, recombination and repair (2,335, 8.18%), translation, ribosomal structure and biogenesis (2,243, 7.86%), posttranslational modification, protein turnover, and chaperones (2,157, 7.56%), carbohydrate transport and metabolism (1,795, 6.29%) and signal transduction mechanisms (1,788, 6.27%).

For further functional prediction and classification, all of the 77,647 unigenes were aligned to the Clusters of Orthologous Groups of proteins (COG) category. Overall, 19.30% of unigenes (14,987) were assigned into 25 COG categories with 28,537 COG functional terms (Figure 4 and Table S3). The categories including transcription (2,455, 8.60%), carbohydrate transport and metabolism (1,795, 6.29%), signal transduction mechanisms (1,788, 6.27%) and secondary metabolites biosynthesis, transport and catabolism (860, 3.01%) were identified, which might be related to response for drought stress in plants.

#### KEGG pathway mapping

To further gain insights into the biological functions and interactions of our unigenes, a pathway-based analysis was performed in the light of the Kyoto Encyclopedia of Genes and Genomes (KEGG) Pathway database which based on the roles in biochemical pathways. There were 23,569 (30.35%) out of the 77,647 unigenes were mapped to 128 KEGG pathways. Among them, 5,056 unigenes (21.45%) were related to metabolic pathways (Ko01100, no maps in KEGG database), 2,460 (10.87%) to biosynthesis of secondary metabolites (Ko01100, no maps in KEGG database). The highest representation with KEGG map was Plant-pathogen interaction (Ko04626, 1,156 unigenes, 4.90%), followed by Plant hormone signal transduction (Ko04075, 1,113 unigenes, 4.72%) and RNA transport (Ko03013, 1,002 unigenes, 4.25%) (Table S4). To be mentioned, 57 core enzymes was detected in the biosynthetic pathway of flavonoid (Ko00941, 228 unigenes) which involved in secondary metabolism under abiotic stresses in plants [36] (Figure S2). Also, all of the core components were found in the circadian rhythm pathway (Ko04712, 192 unigenes), which is crucial for timing of flower and budset in response to the seasonal rhythm of temperature and light length [37], [38] (File S1). *R. soongorica* has been regarded as a C_3_ plant based on the photosynthesis characteristics [39], but all the core genes of C_4_ carbon fixation were found in our transcriptome, surprisingly (Ko00710, 155 unigenes, File S2). Absisic acid (ABA) is a crucial hormone involved in many stress responses [40]. The key enzymes in its biosynthetic and catabolic pathways (Ko00906) and receptor genes (Ko04075) were discovered as well (Table S5 and File S3).

#### Rfam and Pfam analysis

**Figure 5:**
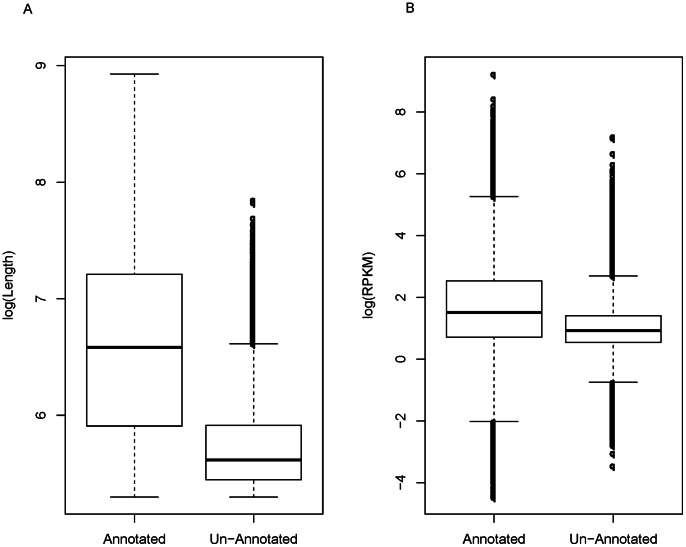
Boxplot distribution of unigenes in annotated and un-annotated unigenes. (A) Length distribution inferred with log(Length). (B) Expression level inferred by log(RPKM) value. Both show significant in ANOVA tests *p* = 2.2e-16.

From Nr, Nt, Swiss-Prot, GO, COG, and KEGG databases, more than half of the unigenes (42,839, 55.17%, mean length was 975 bp, Table 2 and Figure 5A) were annotated, while 34,808 (44.83%) unigenes (mean length was 334 bp, Figure 5A) failed to be annotated. As shown in Figure 5B, the annotated transcripts are significant more abundant in the pool (ANOVA *p* value = 2.2e-16).

To further exploit the potential function of the un-annotated unigenes, Rfam and Pfam analysis were conducted sequentially. Among the un-annotated unigenes, 1,904 showed significant hits (E-value<0.01, Identity >82.30%) with the deposits in the Rfam database, but only 42 were trustable (E-value<1e-5, Identity >84.47%, BLAST coverage >50%, File S4). Furthermore, 173 out of 34,808 un-annotated unigenes contained 92 kinds of Pfam domains (E-value<1e-5), among which reverse transcriptase (RVT_3) and zinc-binding in reverse transcriptase (zf-RVT) domains were highly represented (26 and 19 times, respectively; File S4).

### Functional Genes Related to Drought Adaptation

To screen functional genes related to drought adaptation through three main strategies (drought escape, drought avoidance and drought tolerance; reviewed in [41], [42]), candidate genes from *Arabidopsis* were local BLASTed against 77,647 unigenes in *R. soongorica*. A total of 123 unigenes homologous to 113 *Arabidopsis* candidate genes potentially involved in drought adaptation were identified in our transcriptomic dataset (Table S5). Among them, 46 putative flowering time unigenes were identified to be involved in drought escape (E-value<1e-5) which is a common cost-effective strategy to avoid drought stress in natural populations [41], [42]. A total of 40 unigenes were found to be potentially involved in drought avoidance (E-value<1e-5), which regulate the biogenesis and development of the cuticle (eight unigenes), stomata (six unigenes), and trichomes and root system (26 unigenes). For drought tolerance strategy, the ABA-dependent and -independent pathways have been extensively studied. Totally, 32 unigenes were involved in ABA-dependent pathways, including biosynthetic and catabolic genes, ABA receptors, while eight unigenes were involved in ABA-independent pathway. These genes will be helpful for exploiting the genetic mechanism of how *R. soongorica* adapts to drought natural environment in northern China.

## Discussion

In this study, a large amount of *R. soongorica* transcriptomic unigenes (77,647) were sequenced with Illumina HiSeq™ 2000 platform (Table 1). The N50 length of unigenes was 1,109 bp and the average length was 677 bp; these results were comparable to the recently published plant trancriptomic analysis, such as *Hevea brasiliensis* (N50 = 436 bp, average length = 485 bp [43]) and *Dendrocalamus latiflorus* Munro (N50 = 1,132 bp, average length = 736 bp [44]). Up to date, Trinity is one of most powerful packages prevailed in *de novo* assembly of short reads, especially in dealing with alternative splicesomes and paralogs [33]. A total of 24,271 (31.26%) unigenes were generated as clusters which might correspond to putative alternative spliced transcripts and/or paralogous transcripts (“CL”-unigene). The number of clusters with only two types of “CL”-unigenes (i.e. CL1.Contig1_A and CL1.Contig2_A) was notably high in our dataset (5,662 clusters, Figure S3). A considerable number of clusters with more than ten types of “CL”-unigenes were also found in our dataset (Figure S3). For example, thirteen “CL2023.Contigs” showed high identities (E-value<1e-41, Identity>78%) with *Arabidopsis cryptochrome 1* (*CRY1*) (Table S5, File S5), which plays a crucial role in sensing seasonal change of blue light and UV-A to initiate flower properly [45], [46]. The thirteen contigs can be divided into three types based on sequence divergence with identities less than 90% (File S5). Together with the overrepresented numbers of the clusters with more than two types of “CL”-unigenes, these results suggest that a large amount of paralogous transcripts were presented in our dataset, indicating at least a whole genomic duplicate might happen in the evolutionary history of *R. soongorica*. In addition, three alternative splicing sites were found in the alignment of cluster “CL11.contigs” which contained 32 contigs (File S6), showing that splice-isoforms were also produced by the Trinity assembly. Of course, imperfect assembly of short reads by the Trinity cannot be ruled out.

More than half of the unigenes (42,839, 55.17%) were successfully assigned as annotated genes by BLASTing against with public databases Nr, Nt, Swiss-Prot, GO, COG and KEGG, given the absence of genomic information of *R. soongor*ica (Table 2). Notably, the percentage of annotated is the lowest in among the previous studies with the same sequencing strategy during the last year (58.24 to 78.9%, [44], [47], [48]). One possibility of lacking annotation is due to the technical limitation, such as sequencing depth and read length [49], which was common in the all studies with *de novo* transcriptome analysis [44], [47], [48]. We did find the un-annotated sequences were averagely much shorter than the annotated unigenes (334 bp vs 975 bp, Figure 5A). However, there was still a considerable percentage of unigenes (7.96%, 2,770 of 34,808) with length over 500 bp and reads per kilobase per million reads (RPKM) over three failed to hit any homologs in the known plant species (Figure 5). In addition, there were 173 unigenes contained at least one Pfam domain (E-value<1e-5, File S4), among which two reverse transcriptase domains RVT_3 and zf-RVT were highly represented with 26 and 19 times, respectively. These results suggested that a considerable portion of genes in *R. soongorica* might originate from novel retrotransposon mechanisms [50], [51], which not found in any known genomes yet, and the high frequency of reverse transcriptase genes could be an indispensable part of *R. soongorica* genome (778 Mb, 2n = 22 [13]).

So far, *V. vinifera* was the highest related species with known genome to *R. soongorica* (Figure 2C), but fewer than half of annotated unigenes in *R. soongorica* hit protein sequences in *V. vinifera* (Figure 2C). This is consistent with that these two species were classified into different orders, Caryophyllales (*Tamaraceae*, *Reaumuria*) and Vitales (*Vitaceae*, *Vitis*) [52], [53], and with their low bootstrap value support in the ITS phylogenetic tree (Figure 2D). Therefore, the vast un-annotated unigenes (44.83%, 34,808 of 77,647) could only be novel genes compared with the known genomes and specific in the genome of the relict Tertiary plant *R. soongorica*. Functional and expressional studies by Digital Gene Expression analysis and real-time PCR are needed to further corroborate this hypothesis in detail.

After deep sequencing and exhaustive annotation, this endeavor provided a large amount of unigenes that will facilitate to exploit genetic resources in the functional studies and to identify candidate genes responsible for drought adaptation in *R. soongorica*. Candidate genes out of the 77,647 unigenes in *R. soongorica* involved in three drought adaptation strategies (drought avoidance, drought escape and drought tolerance; reviewed in [41], [42]) were analyzed (Table S5). At least eight unigenes were possibly involved in the formation of the thick cuticle on *R. soongorica* leaf surface (8.3 µm [18]), which plays an important role in regulating the exchange of gases and water in plants and can enhance tolerance to drought [54]. To be mentioned, three unigenes were found as homologs of *HvABCG31*/*Eibi1*, an ATP-binding cassette subfamily G full transporter (also found in KEGG, ko02010), which is essential for the cutin formation and the preservation leaf water in wild barley [55]. Stomata, trichomes and root hairs are crucial for water usefulness and maintenance under drought environment (reviewed in [54], [56]), and the molecular mechanisms of the differentiation of these tissues have been extensively studied in *Arabidopsis* [57]–[59]. *FAMA* is required for the terminal differentiation of guard cells [60]. *Glabra 1* (*GL1*) is an important regulator of trichome initiation in *Arabidopsis* [61]. The development of root hairs and trichomes is regulated by *GL2*, *GL3*, *Enhancer of Glabra 3* (*EGL3*) and *Transparent Testa Glabra 1* (*TTG1*) with similar molecular mechanisms in *Arabidopsis* [62]. Here, a set of unigenes potentially involved in the biogenesis and development of these structures were identified (Table S5), which will facilitate to disentangle the formation of the specific traits such as hollow stomata and deep root system [18] and to further understand the molecular mechanism of drought avoidance strategy in desert plants.

Drought stress can promote plants flowering earlier [63]–[65]. In our field survey, flowering time of *R. soongorica* obviously varied between the populations along a precipitation gradient. The plants in Haishiwan, Gansu province (annual precipitation 600 mm) were still flowering in November, while the plants in Shashichang, Gansu province (precipitation 180 mm) had finished flowering and started to disperse seeds in September (data not shown). We propose that the genes control flowering time might have undergone strong natural selection in *R. soongorica* populations from extreme arid regions. Finally, including all the core components in circadian rhythm in plants KEGG pathway (Ko04712, File S1), 29 out of 51 flowering time genes in *Arabidopsis* can hit their homologous unigenes in *R. soongorica* (Table S5). Further investigating the correlation between the genetic diversity of these candidate genes and the variation of flowering time along drought gradient could shed light on how *R. soongorica* adapts to natural arid environment by drought escape strategy.

The molecular response to drought stress has been studied intensively in model plants, and at least two pathways are suggested to be involved into drought tolerance: ABA-dependent and -independent pathways (reviewed in [66], [67]). In this study, most of the key genes related to ABA biosynthesis, catabolic and receptor complex were identified by KEGG annotation and local BLAST (File S3 and Table S5), such as rate-limiting enzyme *9-cis-epoxycarotenoid dioxygenases* (*NCED*) in the biosynthetic pathway [68], receptor complex components *pyrabactin resistance/PYR1-like/regulatory components of ABA receptor* (*PYR/PYL/RCAR*) [69], *protein phosphatase 2Cs* (*PP2Cs*) to sensor ABA signal [70], and important kinases and transcription factors (i.e. *SNF1-related protein kinase 2s* (*SnRK2s*) [71], *ABRE-binding factor/ABA-responsive elements* (*ABF/AREBs*) [72]). All of these key unigenes may function in ABA signal transduction in response to drought stress as in model plants. Furthermore, several transcription factors like *Dehydration Responsive Element Binding/C-repeat Binding Factors* (*DREB/CBFs*) which are involved in ABA-independent pathways were identified by local BLASTing (Table S5). These genes will help to uncover the molecular basis of physiological responses to drought stress in *R. soongorica*.

**Figure 6:**
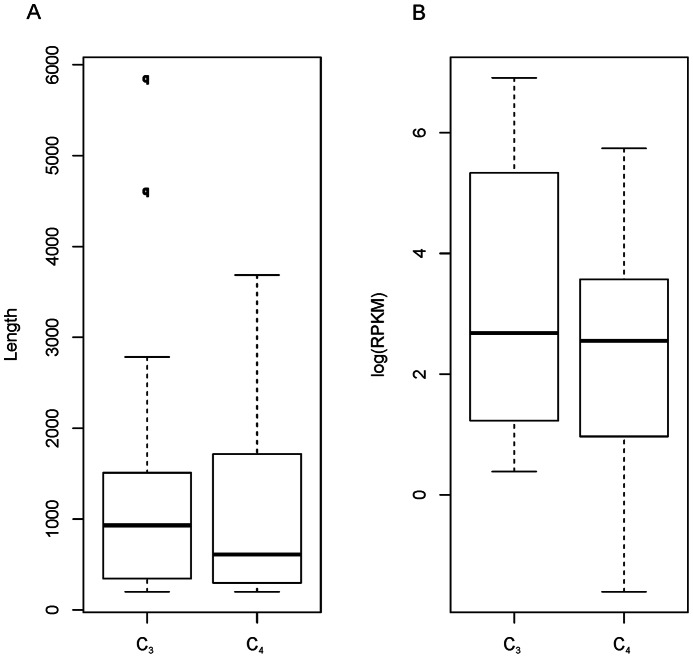
Boxplot distribution of C_3_ and C_4_ unigenes. (A) Length distribution inferred with log(Length). (B) Expression level inferred by log(RPKM) value.

The C_4_ pathway has been acknowledged to be more adaptive than the C_3_ pathway in response to abiotic stresses, such as high temperature, radiation and drought [73], [74]. The C_3_ and C_4_ photosynthesis can occur simultaneously in different leaves within the same plant [75], and the transition between the C_3_ and C_4_ pathways can also occur in some C_3_ plants under some conditions (i.e. *Eleocharis vivipara* [76], *Flaveria brownii* [77]). Nevertheless, the origin of C_4_ pathway and the transition between the C_3_ and C_4_ pathways remained elusive because of the absence of genetic evidence. *R. soongorica* is a C_3_ plant according to its physiological characteristics [32]. In this study, all of the genes encode key enzymes in C_4_ carbon fixation pathway were presented in the transcriptomic dataset from the annotation of KEGG (File S2). The lengths of the C_4_ genes were not statistically different with the C_3_ genes (ANOVA *p* = 0.83), but the C_3_ genes were significantly abundant than C_4_ genes (*p* = 2.7e-05, Figure 6). This is partially concordant to the previous studies which characterized *R. soongorica* as a C_3_ plant [32], [78], [79]. To our knowledge, the present and active of the C_4_ genes in *R. soongorica* might be the first transcriptomic evidence to support that a Tamaricaceae plant could also orchestrate the C_3_ and C_4_ pathways in response to environmental changes [76], [80], [81].

### Conclusion and Perspectives

Desert plants have attracted more and more attentions, because they contain plenty of potential genetic resources to adapt to the extremely harsh conditions in their habitats. In the recent years, transcriptome sequencing became a most powerful and efficient approach to uncover genomic information in non-model organisms [82], [83], few studies (but see [84]) focused on exploitation of the molecular basis of drought adaptation of desert plants. In present study, 77,647 unigenes were generated from a Tertiary relic species *R. soongorica* with the Illumina HiSeq™ 2000 platform and more than half of unigenes has been annotated. At least 123 candidate genes related to drought adaptation were identified by the KEGG annotation and local BLAST, and population genetic study on these candidate genes will help us to better understand the adaptive evolutionary mechanism of *R. soongorica*. Expression and function analysis of the un-annotated unigenes will be also employed to unravel the specific drought adaptation mechanism in *R. soongorica*. These endeavors will advance our knowledge of a dominant plant species coping with the global climate change in the fragile desert ecosystems.

## Materials and Methods

### Ethics Statement

*R. soongorica* is a species widely distributed in Shashichang, Jingtai County, Gansu province and other arid regions, and it is not included into any list of endangered or protected species. Before collecting the samples, an oral permission was got from the local management of forestry after applying with introduction letters of CAREERI (Cold and Arid Regions Environmental and Engineering Research Institute, Chinese Academy of Sciences).

### Plant Materials

Leaves and inflorescences of *R. soongorica* (Figure S4A) were collected in wild field, Shashichang, Jingtai County, Gansu province, northwest of China (37°21′41″N,104°8′11″E), where the average annual precipitation is 180 mm from 1950 to 2000 (http://www.worldclim.org/). Tissues were immediately frozen in liquid nitrogen for later extraction of total RNA. *R. soongorica* seeds from the same place were planted on damp filter papers and incubated at 4°C for 4 days before being placed at 23°C under long-day (16 h light/8 h dark) condition with a photosynthetic photon flux density of 150 µmol m^−2^ s^−1^. Two weeks after germination, seedlings were harvested for RNA isolation (Figure S4B).

### RNA Extraction, Library Preparation and RNA-seq

Total RNA was extracted with E.Z.N.A® Plant RNA Kit (Omega Bio-tek, Doraville, GA, USA). The concentration and quality of each RNA sample were determined by NanoDrop 2000™ micro-volume spectrophotometer (Thermo Scientific, Waltham, MA, USA) and gel electrophoresis. One sample of total RNA was extracted from mature plant tissues including flowers and leaves. Another sample was from whole seedlings including roots, hypocotyls, and cotyledons. The two RNA samples were pooled equally to construct the cDNA library with a final concentration of 610.4 ng/µl.

The poly (A) mRNA was enriched by magnetic Oligo (dT) beads, and then was interrupted into 200–700 nt fragments. Using these short fragments as templates, the first cDNA strand was synthesized by random hexamers primers, followed by the second-stand cDNA synthesis using DNA polymerase I (New England BioLabs) and RNase H (Invitrogen). The short DNA fragments were purified with a QiaQuick PCR extraction kit (QIAGEN Inc., Valencia, CA, USA) for following end repairing and tailing A. Then the DNA fragments were ligated to sequencing adaptors, and the DNA fragments with required length were purified by agarose gel electrophoresis and gathered by PCR amplification. Finally, a paired-end library with insert sizes of 200–700 bp was sequenced using Illumina HiSeq™ 2000 with the average read length of 90 bp. The raw data are available in the ArrayExpress database, E-MTAB-1543 (http://www.ebi.ac.uk/arrayexpress/experiments/E-MTAB-1543/).

### *De novo* Assembly and Expression Profiling

The clean reads were obtained after filtering adapter sequences, reads with 5% ambiguous sequences ‘N’, and low-quality reads (reads with a base quality less than Q20), with a custom PERL script. Then, Trinity, a package consisting of Inchworm, Chrysalis and Butterfly, was used to perform the *de novo* assembly of high-quality clean reads [33]. The command-line parameters used were “–seqType fq –min_contig_length 200–CPU 5–bflyHeapSpaceMax 4G –JM 20G”. Short reads with overlapping sequences were firstly assembled by Inchworm to form the longest contigs without gaps. These contigs were pooled to build into *de* Brujin graphs by Chrysalis. According to the paired end information, Butterfly reconciled the *de* Bruijn graphs and output longer sequences without overlooking the possibility of splice forms and paralogous transcripts. Such sequences which cannot be extended on either end were defined as unigenes. After clustering, the unigenes were divided into clusters (prefix is “CL”) and singletons (prefix is “Unigene”).

RPKM of each unigene was normalized by ERANGE3.1 software to determine the unigene expression profiles [85].

### Functional Annotation

All sequences were annotated by aligning with public protein and nucleotide databases, such as the NCBI Nr, Nt database, the Swiss-Prot protein database, the KEGG database, and the COG with an E-value cutoff of 1e-5. Based on the alignment results, the further annotation analysis with GO terms, which described biological processes, molecular functions and cellular components, was performed by Blast2GO software [35], [86]. The distribution of the gene functions was plotted by WEGO [87].

Then non-coding RNA was retrieved from the Rfam database (http://rfam.sanger.ac.uk/). The sequences excluded non-conding RNAs were BLASTed against Pfam-A database (http://pfam.sanger.ac.uk/) for further seeking functional domains using a hidden Markov model (HMM) algorithm (version 26.0) [88].

### Identification of Putative Candidate Genes Involved in Drought Adaptation

Generally, plants take three main strategies (drought escape, drought avoidance and drought tolerance) to adapt to drought conditions (reviewed in [41], [42]). To uncover the potential candidate genes related to the three strategies in *R. soongorica*, genes in model plant *Arabidopsis* involved in flowering time (drought escape), epidermal development such as stomata, cuticle waxes, trichomes and root hairs (drought avoidance), and ABA and drought stress signals (drought tolerance), were selected to screen the potential orthologs from the unigene dataset by Local BLASTN with E-value cutoff of 1e-5 (ftp://ftp.ncbi.nlm.nih.gov/blast/executables/LATEST-BLAST/).

## Supporting Information

Figure S1 Length distributions of contigs and unigenes. (A) Summary distribution of the lengths of the 173,700 assembled contigs (>100 bp, mean length = 321 bp, N50 = 532 bp). (B) Summary distribution of the lengths of the 77,647 assembled unigenes (>200 bp, mean length = 677 bp, N50 = 1109 bp).
(TIFF)

Figure S2 The flavonoid pathway from KEGG annotation.
(TIF)

Figure S3 The distribution of “CL”-unigenes for *Reaumuria soongorica*. The x-axis represents the number of contigs a “CL”-unigene composed.
(TIF)

Figure S4 The picture of *Reaumuria soongorica*. (A) Adult *R. soongorica*. (B) Two-week old seedlings of *R. soongorica.*
(TIF)

Table S1 List of unigenes with significant BLASTN matches against Nr, Nt, Swiss-Prot and KEGG databases.
(XLSX)

Table S2 The summary of GO annotation for *Reaumuria soongorica*.
(XLSX)

Table S3 The summary of COG annotation for *Reaumuria soongorica.*
(XLSX)

Table S4 The summary of KEGG annotation for *Reaumuria soongorica.*
(XLSX)

Table S5 List of candidate genes involved in drought escape, drought avoidance and drought tolerance in *Reaumuria soongorica* (Unigenes appears multiple times were counted once in article).
(XLSX)

File S1 The unigenes involved in circadian rhythm of *Reaumuria soongorica* from KEGG annotation.
(RAR)

File S2 The unigenes involved in carbon fixation in photosynthetic organisms of *Reaumurica soongorica* from KEGG annotation.
(RAR)

File S3 The unigenes involved in ABA biosynthetic and catabolic pathways and ABA receptors in *Reaumuria soongorica* from KEGG annotaton.
(RAR)

File S4 The Rfam and Pfam annotations of *Reaumuria soongorica* unigenes.
(RAR)

File S5 Multiple sequence alignment of *AtCRY1* and CL2023.Contigs by CLUSTALW.
(RAR)

File S6 Multiple sequence alignment of CL11.Contigs by CLUSTALW.
(RAR)

## References

[1] Manel S, Joost S, Epperson BK, Holderegger R, Storfer A, et al (2010) Perspectives on the use of landscape genetics to detect genetic adaptive variation in the field. Mol Ecol 19: 3760–3772. 20723056 10.1111/j.1365-294X.2010.04717.x

[2] Stapley J, Reger J, Feulner PGD, Smadja C, Galindo J, et al (2010) Adaptation genomics: the next generation. Trends Ecol Evol 25: 705–712. 20952088 10.1016/j.tree.2010.09.002

[3] Liu JL, Li FR, Liu CA, Liu QJ (2012) Influences of shrub vegetation on distribution and diversity of a ground beetle community in a Gobi desert ecosystem. Biodivers Conserv 21: 2601–2619.

[4] Li XR, Zhang P, Su YG, Jia RL (2012) Carbon fixation by biological soil crusts following revegetation of sand dunes in arid desert regions of China: A four-year field study. CATENA 97: 119–126.

[5] Saul-Tcherkas V, Steinberger Y (2011) Soil microbial diversity in the vicinity of a Negev Desert shrub-*Reaumuria negevensis* . Microb Ecol 61: 64–81. 21052657 10.1007/s00248-010-9763-x

[6] Slemnev NN, Gunin PD, Kazantseva TI (1994) On the problem of natural restitution of dominant plants in the ecosystems of the desert zone of Mongolia. Rastit Resur 30: 1–15.

[7] Liu YB, Zhang TG, Li XR, Wang G (2007) Protective mechanism of desiccation tolerance in *Reaumuria soongorica*: Leaf abscission and sucrose accumulation in the stem. Sci China Ser C Life Sci 50: 15–21. 17393078 10.1007/s11427-007-0002-8

[8] Xu L, Wang L, Yue M, Zhao GF, Wang YL (2003) Analysis of grey relatedness between the modular sturcture of *Reaumuria soongorica* population in the desert of Fukang,Xinjiang and the environmental factors. Acta Phytoecologica Sinica 27: 742–748.

[9] Bai YF, Wu JG, Xing Q, Pan QM, Huang JH, et al (2008) Primary production and rain use efficiency across a precipitation gradient on the Mongolia plateau. Ecology 89: 2140–2153. 18724724 10.1890/07-0992.1

[10] Zhou Y, Pei ZQ, Su JQ, Zhang JL, Zheng YR, et al (2012) Comparing soil organic carbon dynamics in perennial grasses and shrubs in a saline-alkaline arid region, northwestern China. PLoS One 7: e42927. 22900067 10.1371/journal.pone.0042927 PMC3416791

[11] Ramadan T (1998) Ecophysiology of salt excretion in the xero-halophyte *Reaumuria hirtella* . New Phytol 139: 273–281.

[12] Gorai M, Neffati M (2007) Germination responses of *Reaumuria vermiculata* to salinity and temperature. Ann Appl Biol 151: 53–59.

[13] Wang XH, Zhang T, Wen ZN, Xiao HL, Yang ZJ, et al (2011) The chromosome number, karyotype and genome size of the desert plant diploid *Reaumuria soongorica* (Pall.) Maxim. Plant Cell Rep 30: 955–964. 21327391 10.1007/s00299-011-1020-3

[14] Yang XP, Rost KT, Lehmkuhl F, Zhu ZD, Dodson J (2004) The evolution of dry lands in northern China and in the Republic of Mongolia since the Last Glacial Maximum. Quaternary Int 118–119: 69–85.

[15] Zhu ZD, Wu Z, Liu S, Di X (1980) An outline of Chinese deserts. Beijing: Science Press.

[16] Yang XP, Scuderi L, Paillou P, Liu ZT, Li HW, et al (2011) Quaternary environmental changes in the drylands of China - A critical review. Quaternary Sci Rev 30: 3219–3233.

[17] Guo ZT, Ruddiman WF, Hao QZ, Wu HB, Qiao YS, et al (2002) Onset of Asian desertification by 22 Myr ago inferred from loess deposits in China. Nature 416: 159–163. 11894089 10.1038/416159a

[18] Liu JQ, Qiu MX, Pu JC, Lu ZM (1982) The typical extreme xerophyte-*Reaumuria soongorica* in the desert of China. Act bot sinica 24: 485–488.

[19] Chong PF, Li Y, Su SP, Gao M, Qiu ZJ (2010) Photosynthetic characteristics and their effect factors of *Reaumuria soongorica* on three geographical populations. Acta Ecol Sin 30: 914–922.

[20] Finkelstein R, Gampala SS, Lynch TJ, Thomas TL, Rock CD (2005) Redundant and distinct functions of the ABA response loci ABA-insensitive(ABI)5 and ABRE-binding factor(ABF)3. Plant Mol Biol 59: 253–267. 16247556 10.1007/s11103-005-8767-2

[21] Bai J, Gong CM, Chen K, Kang HM, Wang G (2009) Examination of antioxidative system’s responses in the different phases of drought stress and during recovery in desert plant *Reaumuria soongorica* (Pall.) Maxim. J Plant Biol 52: 417–425.

[22] Bai J, Gong CM, Wang G, Kang HM (2010) Antioxidative characteristics of *Reaumuria soongorica* under drought stress. Acta Bot Boreali-Occident Sin 30: 2444–2450.

[23] Bai J, Xu DH, Kang HM, Chen K, Wang G (2008) Photoprotective function of photorespiration in *Reaumuria soongorica* during different levels of drought stress in natural high irradiance. Photosynthetica 46: 232–237.

[24] Chong PF, Su SP, Li Y (2011) Comprehensive evaluation of drought resistance of *Reaumuria soongorica* from four geographical populations. Acta Prataculturae Sinica 20: 26–33.

[25] Lv MT, Yang JY, Yang M, Zhang ZR, Ma X (2010) Effect of different drought stress conditions on germination of *Reaumuria soongorica* seeds. Chinese Journal of Grassland 32: 58–63.

[26] Yan QD, Su PX, Gao S (2012) Response of photosynthetic characteristics of C_3_ desert plant *Reaumuria soongorica* and C_4_ desert plant Salsola passerina to different drought degrees. Journal of Desert Research 32: 364–371.

[27] Zeng YJ, Wang YR, Zhuang GH, Yang ZS (2004) Seed germination responses of *Reaumuria soongorica* and *Zygophyllum xanthoxylum* to drought stress and sowing depth. Acta Ecol Sin 24: 1629–1634.

[28] Xu L, Wang YL, Wang XM, Zhang LJ, Yue M, et al (2003) Genetic structure of *Reaumuria soongorica* population in Fukang Desert, Xinjiang and its relationship with ecological factors. Acta Bot Sin 45: 787–794.

[29] Li XL, Chen J, Wang G (2008) Spatial autocorrelation analysis of ISSR genetic variation of *Reaumuria soongorica* population in northwest of China. Journal of Desert Research 28: 468–472.

[30] Qian ZQ, Xu L, Wang YL, Yang J, Zhao GF (2008) Ecological genetics of *Reaumuria soongorica* (Pall.) Maxim. population in the oasis-desert ecotone in Fukang, Xinjiang, and its implications for molecular evolution. Biochem Syst Ecol 36: 593–601.

[31] Li ZH, Chen J, Zhao GF, Guo YP, Kou YX, et al (2012) Response of a desert shrub to past geological and climatic change: A phylogeographic study of *Reaumuria soongarica* (Tamaricaceae) in western China. J Syst Evol 50: 351–361.

[32] Ma JY, Chen T, Qiang WY, Wang G (2005) Correlations between foliar stable carbon isotope composition and environmental factors in desert plant *Reaumuria soongorica* (Pall.) Maxim. J Integr Plant Biol 47: 1065–1073.

[33] Grabherr MG, Haas BJ, Yassour M, Levin JZ, Thompson DA, et al (2011) Full-length transcriptome assembly from RNA-Seq data without a reference genome. Nat Biotechnol 29: 644–652. 21572440 10.1038/nbt.1883 PMC3571712

[34] Ashburner M, Ball CA, Blake JA, Botstein D, Butler H, et al (2000) Gene Ontology: tool for the unification of biology. Nat Genet 25: 25–29. 10802651 10.1038/75556 PMC3037419

[35] Conesa A, Gotz S, Garcia-Gomez JM, Terol J, Talon M, et al (2005) Blast2GO: a universal tool for annotation, visualization and analysis in functional genomics research. Bioinformatics 21: 3674–3676. 16081474 10.1093/bioinformatics/bti610

[36] Winkel-Shirley B (2002) Biosynthesis of flavonoids and effects of stress. Curr Opin Plant Biol 5: 218–223. 11960739 10.1016/s1369-5266(02)00256-x

[37] Mouradov A, Cremer F, Coupland G (2002) Control of flowering time interacting pathways as a basis for diversity. The Plant Cell Online 14: S111–S130. 10.1105/tpc.001362 PMC151251 12045273

[38] Böhlenius H, Huang T, Charbonnel-Campaa L, Brunner AM, Jansson S, et al (2006) CO/FT regulatory module controls timing of flowering and seasonal growth cessation in trees. Science 312: 1040–1043. 16675663 10.1126/science.1126038

[39] Xu DH, Su PX, Zhang RY, Li HL, Zhao L, et al (2010) Photosynthetic parameters and carbon reserves of a resurrection plant *Reaumuria soongorica* during dehydration and rehydration. Plant Growth Regul 60: 183–190.

[40] Leung J, Giraudat J (1998) Abscisic acid signal transduction. Annu Rev Plant Physiol Plant Mol Biol 49: 199–222. 15012233 10.1146/annurev.arplant.49.1.199

[41] Chaves MM, Maroco JP, Pereira JS (2003) Understanding plant responses to drought - from genes to the whole plant. Funct Plant Biol 30: 239–264. 10.1071/FP02076 32689007

[42] Mckay JK, Richards J, Mitchell-Olds T (2003) Genetics of drought adaptation in *Arabidopsis* thaliana: I. Pleiotropy contributes to genetic correlations among ecological traits. Mol Ecol 12: 1137–1151. 12694278 10.1046/j.1365-294x.2003.01833.x

[43] Xia ZH, Xu HM, Zhai JL, Li DJ, Luo HL, et al (2011) RNA-Seq analysis and *de novo* transcriptome assembly of *Hevea brasiliensis* . Plant Mol Biol 77: 299–308. 21811850 10.1007/s11103-011-9811-z

[44] Liu MY, Qiao GR, Jiang J, Yang H, Xie LH, et al (2012) Transcriptome sequencing and *de novo* analysis for Ma Bamboo (*Dendrocalamus latiflorus* Munro) using the Illumina platform. PLoS One 7: e46766. 23056442 10.1371/journal.pone.0046766 PMC3463524

[45] El-Assal SED, Alonso-Blanco C, Peeters AJM, Raz V, Koornneef M (2001) A QTL for flowering time in *Arabidopsis* reveals a novel allele of CRY2. Nat Genet 29: 435–440. 11726930 10.1038/ng767

[46] Mas P, Devlin PF, Panda S, Kay SA (2000) Functional interaction of phytochrome B and cryptochrome 2. Nature 408: 207–211. 11089975 10.1038/35041583

[47] Huang LL, Yang X, Sun P, Tong W, Hu SQ (2012) The first Illumina-based *de novo* transcriptome sequencing and analysis of Safflower Flowers. PLoS One 7: e38653. 22723874 10.1371/journal.pone.0038653 PMC3378585

[48] Xu DL, Long H, Liang JJ, Zhang J, Chen X, et al (2012) *De novo* assembly and characterization of the root transcriptome of *Aegilops variabilis* during an interaction with the cereal cyst nematode. BMC Genomics 13: 133. 22494814 10.1186/1471-2164-13-133 PMC3439707

[49] Novaes E, Drost DR, Farmerie WG, Pappas GJ Jr, Grattapaglia D, et al (2008) High-throughput gene and SNP discovery in *Eucalyptus grandis*, an uncharacterized genome. BMC Genomics 9: 312. 18590545 10.1186/1471-2164-9-312 PMC2483731

[50] Flavell AJ (1995) Retroelements, reverse transcriptase and evolution. Comp Biochem Physiol B Biochem Mol Biol 110: 3–15. 7532085 10.1016/0305-0491(94)00122-b

[51] Xiong Y, Eickbush TH (1990) Origin and evolution of retroelements based upon their reverse-transcriptase sequences. EMBO J 9: 3353–3362. 1698615 10.1002/j.1460-2075.1990.tb07536.x PMC552073

[52] Soltis DE, Soltis PS, Chase MW, Mort ME, Albach DC, et al (2000) Angiosperm phylogeny inferred from 18S rDNA, rbcL, and atpB sequences. Bot J Linn Soc 133: 381–461.

[53] The Angiosperm Phylogeny Group (2003) An update of the Angiosperm Phylogeny Group classification for the orders and families of flowering plants: APG II. Bot J Linn Soc 141: 399–436.

[54] Yang J, Isabel Ordiz M, Jaworski JG, Beachy RN (2011) Induced accumulation of cuticular waxes enhances drought tolerance in *Arabidopsis* by changes in development of stomata. Plant Physiol Biochem 49: 1448–1455. 22078383 10.1016/j.plaphy.2011.09.006

[55] Chen GX, Komatsuda T, Ma JF, Nawrath C, Pourkheirandish M, et al (2011) An ATP-binding cassette subfamily G full transporter is essential for the retention of leaf water in both wild barley and rice. Proc Natl Acad Sci U S A 108: 12354–12359. 21737747 10.1073/pnas.1108444108 PMC3145689

[56] Ishida T, Kurata T, Okada K, Wada T (2008) A genetic regulatory network in the development of trichomes and root hairs. Annu Rev Plant Biol 59: 365–386. 18257710 10.1146/annurev.arplant.59.032607.092949

[57] Pillitteri LJ, Sloan DB, Bogenschutz NL, Torii KU (2006) Termination of asymmetric cell division and differentiation of stomata. Nature 445: 501–505. 17183267 10.1038/nature05467

[58] Schellmann S, Hulskamp M (2005) Epidermal differentiation: trichomes in *Arabidopsis* as a model system. Int J Dev Biol 49: 579–584. 16096966 10.1387/ijdb.051983ss

[59] Bruex A, Kainkaryam RM, Wieckowski Y, Kang YH, Bernhardt C, et al (2012) A gene regulatory network for root epidermis cell differentiation in *Arabidopsis* . PLoS Genet 8: e1002446. 22253603 10.1371/journal.pgen.1002446 PMC3257299

[60] Ohashi-Ito K, Bergmann DC (2006) *Arabidopsis* FAMA controls the final proliferation/differentiation switch during stomatal development. Plant Cell 18: 2493–2505. 17088607 10.1105/tpc.106.046136 PMC1626605

[61] Schnittger A, Jurgens G, Hulskamp M (1998) Tissue layer and organ specificity of trichome formation are regulated by GLABRA1 and TRIPTYCHON in *Arabidopsis* . Development 125: 2283–2289. 9584127 10.1242/dev.125.12.2283

[62] Tominaga-Wada R, Ishida T, Wada T (2011) New Insights into the Mechanism of Development of *Arabidopsis* Root Hairs and Trichomes. Int Rev Cel Mol Bio 286: 67–106. 10.1016/B978-0-12-385859-7.00002-1 21199780

[63] Fox GA (1990) Drought and the evolution of flowering time in desert annuals. Am J Bot 77: 1508–1518.

[64] Franks SJ, Sim S, Weis AE (2007) Rapid evolution of flowering time by an annual plant in response to a climate fluctuation. Proc Natl Acad Sci U S A 104: 1278–1282. 17220273 10.1073/pnas.0608379104 PMC1783115

[65] Nevo E, Fu YB, Pavlicek T, Khalifa S, Tavasi M, et al (2012) Evolution of wild cereals during 28 years of global warming in Israel. Proc Natl Acad Sci U S A 109: 3412–3415. 22334646 10.1073/pnas.1121411109 PMC3295258

[66] Shinozaki K, Yamaguchi-Shinozaki K (2007) Gene networks involved in drought stress response and tolerance. J Exp Bot 58: 221–227. 17075077 10.1093/jxb/erl164

[67] Xiong LM, Schumaker KS, Zhu JK (2002) Cell signaling during cold, drought, and salt stress. Plant Cell 14: S165–S183. 12045276 10.1105/tpc.000596 PMC151254

[68] Nambara E, Marion-Poll A (2005) Abscisic acid biosynthesis and catabolism. Annu Rev Plant Biol 56: 165–185. 15862093 10.1146/annurev.arplant.56.032604.144046

[69] Gonzalez-Guzman M, Pizzio GA, Antoni R, Vera-Sirera F, Merilo E, et al (2012) *Arabidopsis* PYR/PYL/RCAR receptors play a major role in Quantitative regulation of stomatal aperture and transcriptional response to abscisic acid. The Plant Cell Online 24: 2483–2496. 10.1105/tpc.112.098574 PMC3406898 22739828

[70] Ma Y, Szostkiewicz I, Korte A, Moes D, Yang Y, et al (2009) Regulators of PP2C phosphatase activity function as abscisic acid sensors. Science 324: 1064–1068. 19407143 10.1126/science.1172408

[71] Fujita Y, Nakashima K, Yoshida T, Katagiri T, Kidokoro S, et al (2009) Three SnRK2 protein kinases are the main positive regulators of abscisic acid signaling in response to water stress in *Arabidopsis* . Plant Cell Physiol 50: 2123–2132. 19880399 10.1093/pcp/pcp147

[72] Kim SY (2006) The role of ABF family bZIP class transcription factors in stress response. Physiol Plant 126: 519–527.

[73] Taiz L, Zeiger E (1991) Plant Physiology. Redwood City: The Benjamin/Cammings Publishing Company, Inc.

[74] Raven PH, Evert RF, Eichhorn SE (1992) Biology of Plants. New York: Worth Publishers, Inc.

[75] Raghavendra AS, Rajendrudu G, Das VSR (1978) Simultaneous occurrence of C_3_ and C_4_ photosyntheses in relation to leaf position in *Mollugo nudicaulis* . Nature 273: 143–144.

[76] Ueno O (1998) Induction of Kranz anatomy and C_4_-like biochemical characteristics in a submerged amphibious plant by abscisic acid. Plant Cell 10: 571–583. 9548983 10.1105/tpc.10.4.571 PMC144017

[77] Cheng SH, Demoore B, Wu JR, Edwards GE, Ku MSB (1989) Photosynthetic plasticity in *Flaveria Brownii* growth irradiance and the expression of C_4_ photosynthesis. Plant Physiol 89: 1129–1135. 16666675 10.1104/pp.89.4.1129 PMC1055986

[78] Sage RF, Christin PA, Edwards EJ (2011) The C_4_ plant lineages of planet Earth. J Exp Bot 62: 3155–3169. 21414957 10.1093/jxb/err048

[79] Sage RF, Sage TL, Kocacinar F (2012) Photorespiration and the evolution of C_4_ photosynthesis. Annu Rev Plant Biol 63: 17.11–17.29. 10.1146/annurev-arplant-042811-105511 22404472

[80] Brautigam A, Kajala K, Wullenweber J, Sommer M, Gagneul D, et al (2011) An mRNA blueprint for C_4_ photosynthesis derived from comparative transcriptomics of closely related C_3_ and C_4_ species. Plant Physiol 155: 142–156. 20543093 10.1104/pp.110.159442 PMC3075794

[81] Williams BP, Aubry S, Hibberd JM (2012) Molecular evolution of genes recruited into C_4_ photosynthesis. Trends Plant Sci 17: 213–220. 22326564 10.1016/j.tplants.2012.01.008

[82] Wang Z, Gerstein M, Snyder M (2009) RNA-Seq: a revolutionary tool for transcriptomics. Nat Rev Genet 10: 57–63. 19015660 10.1038/nrg2484 PMC2949280

[83] Ekblom R, Galindo J (2011) Applications of next generation sequencing in molecular ecology of non-model organisms. Heredity (Edinb) 107: 1–15. 21139633 10.1038/hdy.2010.152 PMC3186121

[84] Zhou YD, Gao FD, Liu RM, Feng JD, Li HD (2012) *De novo* sequencing and analysis of root transcriptome using 454 pyrosequencing to discover putative genes associated with drought tolerance in *Ammopiptanthus mongolicus* . BMC Genomics 13: 266. 22721448 10.1186/1471-2164-13-266 PMC3407029

[85] Mortazavi A, Williams BA, Mccue K, Schaeffer L, Wold B (2008) Mapping and quantifying mammalian transcriptomes by RNA-Seq. Nature Methods 5: 621–628. 18516045 10.1038/nmeth.1226 PMC13303166

[86] Conesa A, Gotz S (2008) Blast2GO: A comprehensive suite for functional analysis in plant genomics. Int J Plant Genomics 2008: 619832. 18483572 10.1155/2008/619832 PMC2375974

[87] Ye J, Fang L, Zheng H, Zhang Y, Chen J, et al (2006) WEGO: a web tool for plotting GO annotations. Nucleic Acids Res 34: W293–W297. 16845012 10.1093/nar/gkl031 PMC1538768

[88] Punta M, Coggill PC, Eberhardt RY, Mistry J, Tate J, et al (2012) The Pfam protein families database. Nucleic Acids Res 40: D290–D301. 22127870 10.1093/nar/gkr1065 PMC3245129

