# Correction: Transcriptomic Analysis of a Tertiary Relict Plant, Extreme Xerophyte *Reaumuria soongorica* to Identify Genes Related to Drought Adaptation

**DOI:** 10.1371/annotation/46606f45-d2bb-4531-99b2-52136f921d21

**Published:** 2013-12-17

**Authors:** Yong Shi, Xia Yan, Pengshan Zhao, Hengxia Yin, Xin Zhao, Honglang Xiao, Xinrong Li, Guoxiong Chen, Xiao-Fei Ma

In the Funding section, the grant number from the funder Key Project of Chinese National Programs for Fundamental Research and Development was listed incorrectly. The correct grant number is: 2013CB429906. 

