# Supplementary figures and images for: Transcriptomic Analysis of a Tertiary Relict Plant, Extreme Xerophyte Reaumuria soongorica to Identify Genes Related to Drought Adaptation
Source: PLoS One. 2013 May 23;8(5):e63993. doi: 10.1371/journal.pone.0063993 (PMC3662755; doi:10.1371/journal.pone.0063993)

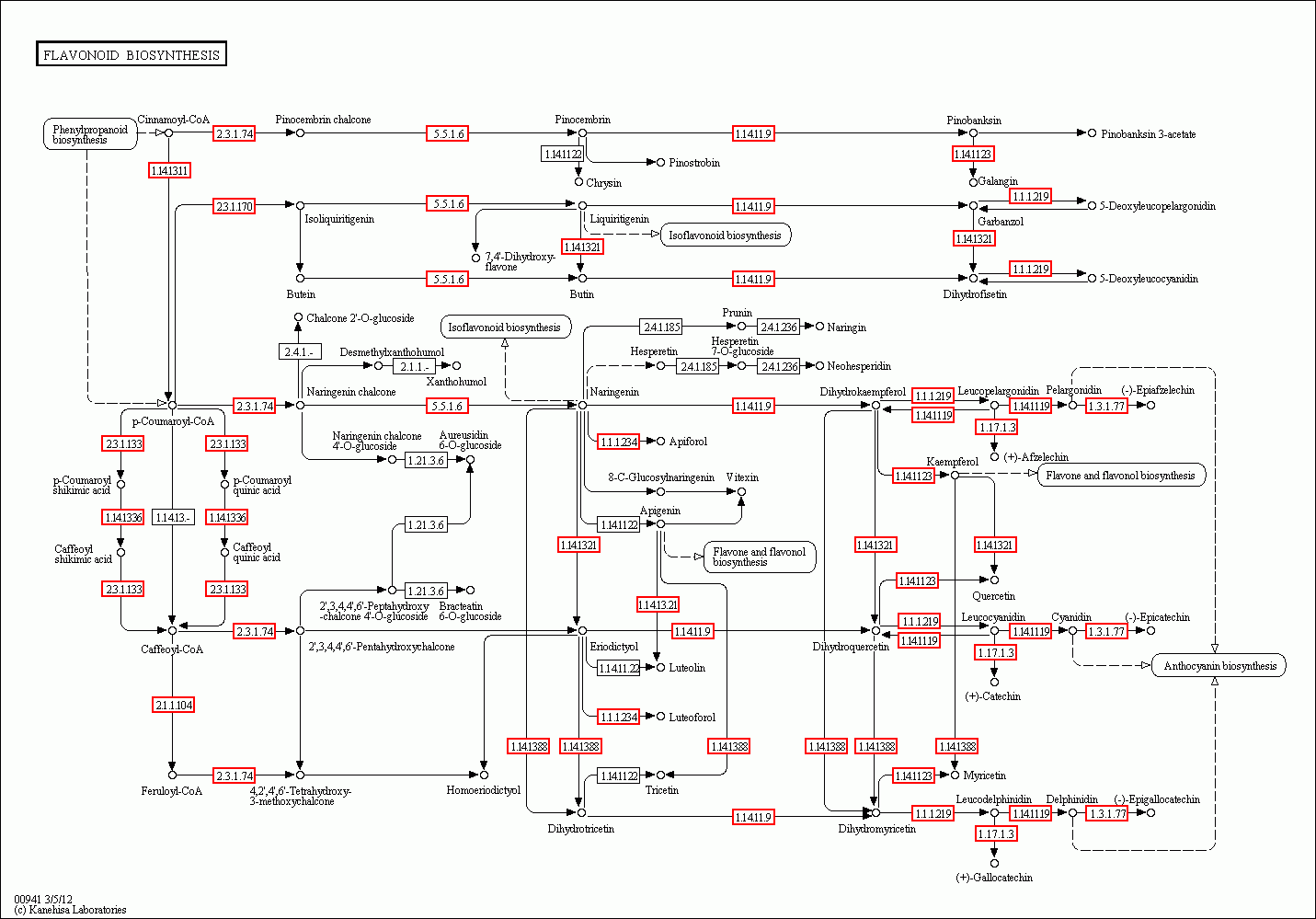

Supplement: Figure S2 — The flavonoid pathway from KEGG annotation. (TIF) [file pone.0063993.s002.tif]

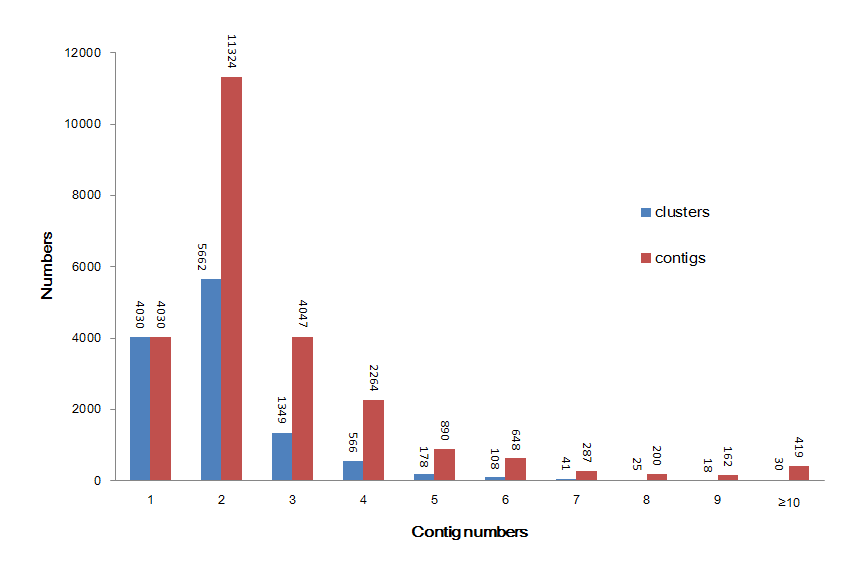

Supplement: Figure S3 — The distribution of “CL”-unigenes for Reaumuria soongorica. The x-axis represents the number of contigs a “CL”-unigene composed. (TIF) [file pone.0063993.s003.tif]

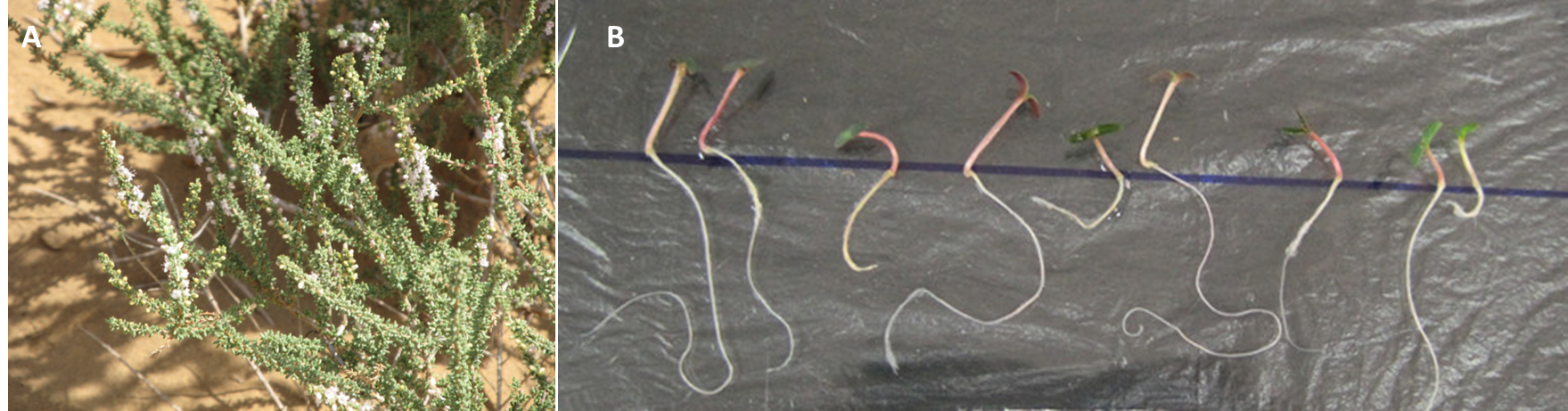

Supplement: Figure S4 — The picture of Reaumuria soongorica. (A) Adult R. soongorica. (B) Two-week old seedlings of R. soongorica. (TIF) [file pone.0063993.s004.tif]
